# Life satisfaction and subjective well-being in urban slums of Gorakhpur, India: psychometric validation of the satisfaction with life scale (SWLS) and socio-demographic assessment

**DOI:** 10.1017/S1463423626100991

**Published:** 2026-03-06

**Authors:** U. Venkatesh, Arshad Ahmed, Ashoo Grover, Om Prakash Bera, Anand Mohan Dixit, Hari Shanker Joshi

**Affiliations:** 1 Department of Community & Family Medicine, All India Institute of Medical Sciences, Gorakhpur, India; 2 Indian Council of Medical Research, New Delhi, India; 3 Global Health Advocacy Incubator (GHAI), Washington, DC, USA; 4 Indian Council of Medical Research, Regional Medical Research Centre, Gorakhpur, India

**Keywords:** Life satisfaction, psychometric validation, subjective well-Being, SWLS, urban slums

## Abstract

**Background:**

Life satisfaction, a core component of subjective well-being, has not been comprehensively explored among urban slum populations. This study aimed to psychometrically assess the Satisfaction with Life Scale (SWLS) and examine socio-demographic correlates of life satisfaction among adults in Gorakhpur, India.

**Methods:**

A cross-sectional study was conducted among 406 participants (52.5% male, 47.5% female) selected through multistage random sampling across eight urban slums in Gorakhpur. Eligible individuals were aged 18 years or above and residents of the selected slum areas. Data were collected using a pre-validated version of the SWLS and a structured socio-demographic questionnaire, administered via the EpiCollect5 through face-to-face interviews. Descriptive and comparative analyses were used to assess group differences across socio-demographic variables.

**Results:**

The SWLS showed good internal consistency (*α* = 0.842) and satisfactory inter-item correlations (*r* = 0.375–0.654, *p* < 0.01). Males reported significantly higher life satisfaction than females, particularly among married and cohabiting individuals (27.30 vs. 23.75, *p* = 0.001) and non-vegetarian consumers (27.28 vs. 24.25, *p* < 0.001). Participants from joint families showed higher satisfaction than those in nuclear households (26.79 vs. 20.29, *p* = 0.011). Women aged 56-65 had the lowest satisfaction scores (14.50 ± 0.71), with half reporting neutrality or dissatisfaction.

**Conclusion:**

The findings highlight the importance of gender, family structure and dietary habits as key socio-cultural correlates of life satisfaction in urban slum communities. The presence of moderate satisfaction levels despite material hardship highlights the need for context-sensitive well-being frameworks and community-informed interventions in similar low-resource settings.

## Introduction

Life satisfaction is a central component of subjective well-being, which reflects an individual’s cognitive appraisal of his or her overall quality of life relative to personally meaningful standards (Diener *et al.*, 1985; Joshanloo and Jovanović, 2021). Distinct from transient affect, it represents a stable evaluative dimension of well-being, one that correlates strongly with physical health, psychological resilience and social functioning (Diener and Chan, 2011; Lyubomirsky *et al.*, 2005; Steptoe *et al.*, 2015). The measurement of life satisfaction has therefore gained prominence among psychologists and public-health researchers seeking to link individual perceptions of well-being with broader socioeconomic and environmental realities (Das *et al.*, 2020; Maccagnan *et al.*, 2019; Veenhoven, 2008).

Despite years of global research, there is still a very limited understanding of life satisfaction in contexts of chronic deprivation. Most comparative surveys, such as the Gallup World Poll, are dominated by data from high-income or middle-class populations (Jan-Emmanuel and Krekel, 2020). Yet, about one-quarter of world’s urban residents live in informal settlements characterized by insecure tenure, overcrowding and restricted access to basic amenities (UN-Habitat, 2003; Ezeh *et al.*, 2017). In these contexts, well-being is determined by material deprivation, but also by adaptive social processes like trust, social cohesion and community participation that shape resilience and perceptions of life quality (Forrest and Kearns, 2001; Kawachi and Berkman, 2014; Biswas-Diener and Diener, 2001; Howell and Howell, 2008).

Recent empirical evidence underlines the complexity of wellbeing under adversity. For example, Biswas-Diener and Diener (2001) assessed life satisfaction among residents of six slums in Kolkata, India. Compared to a metropolitan comparison group, global satisfaction scores were unexpectedly high, implying psychological adaptation to poverty. Again, Sulkers and Loos (2022) and Humble *et al.* (2023) demonstrated that SWB was significantly and positively correlated with neighbourhood social cohesion in Delhi’s informal settlements, especially if residents had a say in the choice of settlement. Parallel findings outside India, such as Matsuguma (2025) study in Philippine slums, revealed that active use of character strengths predicted flourishing despite high distress, supporting dual-factor models of mental health (Keyes, 2002; Niemiec, 2023). These studies highlight that even amid material hardship, psychosocial assets cohesion, trust, agency and strengths use can sustain well-being. Nonetheless, they also reveal methodological and conceptual limitations: limited psychometric validation of key scales, small or gender-skewed samples and scarce integration of socio-demographic determinants beyond income.

Building upon this emerging scholarship, present study advances the literature in three significant ways. First, while previous Indian studies have largely relied on global or domain-specific satisfaction measures (Sulkers and Loos, 2022; Humble *et al.*, 2023), present research employs SWLS (Diener *et al.*, 1985) to provide a psychometrically rigorous assessment. Given that standard scales developed in Western, Educated, Industrialized, Rich and Democratic (WEIRD) contexts may not directly transfer to low-literacy, collectivist and resource-constrained environments, validating SWLS among slum populations is both methodologically and ethically essential (Agnihotri *et al.*, 2010; Skevington *et al.*, 2004). Second, this study examines multiple socio-demographic and behavioural predictors including age, gender, marital status, family structure, socioeconomic status and dietary habits to capture the complex social ecology of life satisfaction in urban poverty (Howell and Howell, 2008). Third, unlike earlier research focused on metropolitan megacities, this investigation is situated in Gorakhpur, Uttar Pradesh a medium-sized flood-prone city in the Indo-Gangetic Plain thereby extending inquiry into understudied peri-urban regions with distinct socio-environmental stressors (Khilnani and Tiwari, 2018; Ray, 2017).

A deeply uneven process of urbanization in India sets the broader context. While the country has achieved sustained economic growth, wide inequalities remain: more than 60 million people reside in informal settlements that are without proper sanitation, potable water and security of tenure (Abdulhadi *et al.*, 2024; Census of India, 2011). Overlapping vulnerabilities-economic insecurity, health risks and social exclusion-endanger subjective well-being among residents in such areas (Ray, 2017; Vásquez-Vera *et al.*, 2017). Yet community cohesion and cultural coping practices may mitigate such influences, implying that poverty in and of itself cannot be a singular factor for disparities in life-satisfaction.

The present study, therefore, attempts (1) to examine internal consistency and construct validity of SWLS among adults living in urban slums of Gorakhpur, Uttar Pradesh and (2) to identify socio-demographic correlates of life satisfaction in this population. More precisely, following analysis investigates influence of age, gender, marital status, family structure, socioeconomic status and dietary patterns relate on variation in life satisfaction.

By integrating psychometric validation with social-determinants analysis, study addresses critical empirical and conceptual gaps left by earlier work in Kolkata (Sulkers and Loos, 2022), Delhi (Humble *et al.*, 2023) and other Asian slum contexts (Matsuguma, 2025). In doing so, it contributes a contextually grounded understanding of subjective well-being in a socioeconomically and environmentally vulnerable urban setting of northern India.

## Methods

### Study design

This cross-sectional quantitative study was conducted between 2023 and 2024 as part of a larger mixed-methods research initiative focused on evaluating physical, mental and social well-being in urban slum populations. The present study specifically aimed to validate SWLS and explore socio-demographic determinants of life satisfaction among adults living in urban slums of Gorakhpur, Uttar Pradesh, India.

### Study area

**Figure 1.**
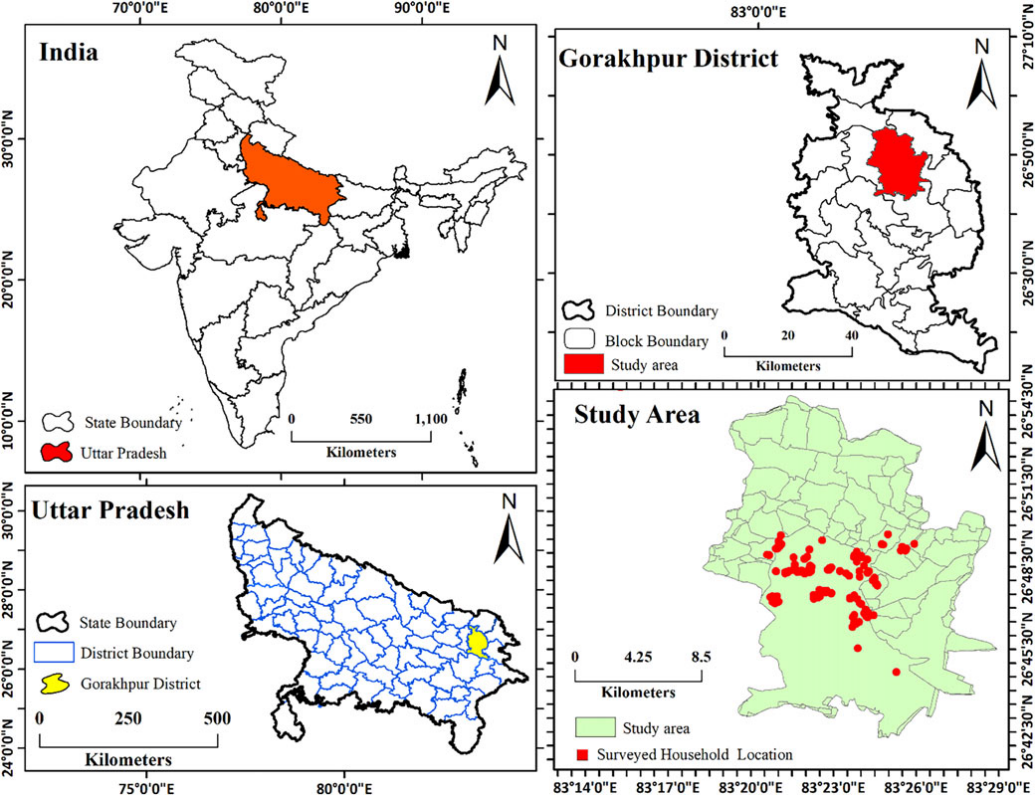
Location of the study area.

Gorakhpur Urban Agglomeration (U.A.) consists of a municipal corporation area along with surrounding Air Force zones, measuring about 142.13 sq. km. The city is governed under the Gorakhpur Municipal Corporation and is part of the Gorakhpur Metropolitan Region (Figure 1). As per Census 2011, the city had a population of 673,446, a sex ratio of 903 females per 1,000, and an average literacy rate of 83.91% with 88.67% for males and 78.65% females (Census of India, 2011). Based on recent demographic projections, estimated population of Gorakhpur city is approximately 983,000, whereas metropolitan population is approximately 1.01 million. Although significant urban growth has occurred and major development initiatives such as PMAY-Urban and SBM-U 2.0 are being implemented, field surveys done in 2023-24 and municipal records revealed that most of settlements still show slum-like conditions, marked by overcrowding, lack of proper sanitation and inadequate access to primary healthcare. A summary of available demographic and infrastructural characteristics of study area has been presented in Table S1.

### Participants

The study was conducted among adults (≥ 18 years) residing in urban slum communities of Gorakhpur. Inclusion criteria consisted of permanent residents who gave informed consent and were available for follow-up interviews. Since this survey was part of a larger digital health research project, only individuals possessing an Android phone were selected to enable communication in future phases of broader project. This did not affect administration, scoring, or interpretation of the result of SWLS and was unrelated to participants’ well-being status. Terminal illness, cognitive impairment and other conditions that precluded ability to participate in an interview excluded persons from this study.

### Sampling and data collection

A multistage random sampling technique was used in selecting eight urban slums in each of the four city zones: North, South, East and West. Households were randomly selected from within each slum and one eligible adult was enrolled from each household. An initial target sample size of 480 participants (30 per slum over two planned survey rounds: 30 × 8 × 2 = 480) was determined based on prior feasibility studies in similar low-resource urban contexts (Billingham *et al.*, 2013) in order to ensure adequate power for psychometric validation of SWLS. The ‘×2’ represented two planned survey rounds at baseline and follow-up under broader project framework.

According to guidelines for psychometrically sound factor analysis, an item-to-participant ratio of at least 1:30 is sufficient (Comrey and Lee, 2013; MacCallum *et al.*, 1999). In view of its five-item composition, SWLS required a minimum of 150–250 participants; sample size achieved therefore had robust statistical power for reliability and validity. This sample size of 406 was reduced from 480 because of non-response and elimination during data cleaning; this exclusion did not compromise representation across city zones. Data were collected through face-to-face interviews conducted by trained fieldworkers, who provided assistance to participants facing literacy challenges.

## Instruments

### Socio-demographic questionnaire

A structured questionnaire was developed to capture key background variables such as age, gender, marital status, type of family (nuclear or joint), religion and socioeconomic status (assessed using revised Kuppuswamy socioeconomic scale) (Ananthan, 2021). In addition, information on dietary habits (vegetarian or non-vegetarian) was collected, as diet type in India is closely linked with cultural identity, socioeconomic status and nutritional well-being factors that may influence subjective life satisfaction (Huang and Zhao, 2020; Appadurai, 1981; Green *et al.*, 2016). These variables were selected based on their established relevance to subjective well-being, especially in low-and middle-income country contexts (Purgato *et al.*,, 2018) and enabled stratified analyses of SWLS outcomes.

### Satisfaction with life scale (SWLS)

Life satisfaction, representing cognitive dimension of subjective well-being, was assessed using SWLS developed by (Diener *et al.*, 1985). This instrument comprises five items that evaluate global cognitive judgements of one’s life satisfaction: (1) ‘In most ways my life is close to my ideal’; (2) ‘The conditions of my life are excellent’; (3) ‘I am satisfied with my life’; (4) ‘So far, I have gotten the important things I want in life’; (5) ‘If I could live my life over, I would change almost nothing’. Each item is rated on a 7-point Likert scale ranging from 1 (strongly disagree) to 7 (strongly agree), producing a composite score ranging from 5 to 35. Higher scores denote greater life satisfaction. The rational for use of SWLS is twofold: first, its brief from along with strong psychometric performance across diverse populations; second, its conceptual clarity in isolation of cognitive-evaluative aspect of subjective well-being, independent of affective states (Pavot and Diener, 2008).

### Administration and comprehension procedures

The original SWLS was administered in English; all participants were interviewed by trained bilingual field investigators who read each question aloud in Hindi, predominant local language. This approach assisted participants with limited literacy while preserving original meaning of each item. Interviewers had been trained to provide neutral and consistent delivery, providing brief explanations only when necessary to avoid misunderstanding. This standardized procedure ensured conceptual consistency, linguistic clarity and cultural appropriateness in responses across study population.

### Data analysis

To analyse differences in SWLS scores according to socio-demographic variables such as gender, age group, marital status, type of family, education and occupation of head of household, socioeconomic status (as per Kuppuswamy scale) (Ananthan, 2021) and dietary habits, independent samples t-tests and one-way ANOVA were conducted. Descriptive statistics were computed for each SWLS item. Inter-item relationships were assessed using Pearson’s correlation coefficients, while internal consistency was evaluated using Cronbach’s alpha. To assess item-level psychometric properties, corrected item-total correlations, skewness and kurtosis were examined. Categorical distributions and percentile ranks of SWLS scores were calculated across gender and age groups.

## Results

### Socio-demographic characteristics

A total of 406 adults from selected urban slums in Gorakhpur district participated in the survey. As shown in Table 1, just over half of the respondents were male (52.5%). The majority were married and living with their spouses (70.9%) and most lived in joint family households (82.0%). Based on Kuppuswamy Socio-Economic Scale (Ananthan, 2021), a large proportion of participants (70.2%) were classified in ‘Upper Lower’ socioeconomic category. In terms of religious affiliation, sample was predominantly Hindu (94.1%), with Muslims comprising a smaller share (5.9%).

**Table 1.** Socio-demographic characteristics of the study participants (*N* = 406)

Characteristic	Category	**Frequency (*n*)**	Percentage (%)
Gender	Male	213	52.5
	Female	193	47.5
Marital status	Never married	106	26.1
	Married and Cohabiting	288	70.9
	Married but Not Cohabiting	10	2.5
	Divorced/Widowed	2	0.5
Type of family	Joint	333	82.0
	Nuclear	73	18.0
Socio-economic Status (Kuppuswamy scale)	Upper	0	0.0
	Upper Middle	5	1.2
	Lower Middle	28	6.9
	Upper Lower	285	70.2
	Lower	88	21.7
Religion	Hindu	382	94.1
	Muslim	24	5.9
	Christian	0	0.0
	Others	0	0.0

**Figure 2.**
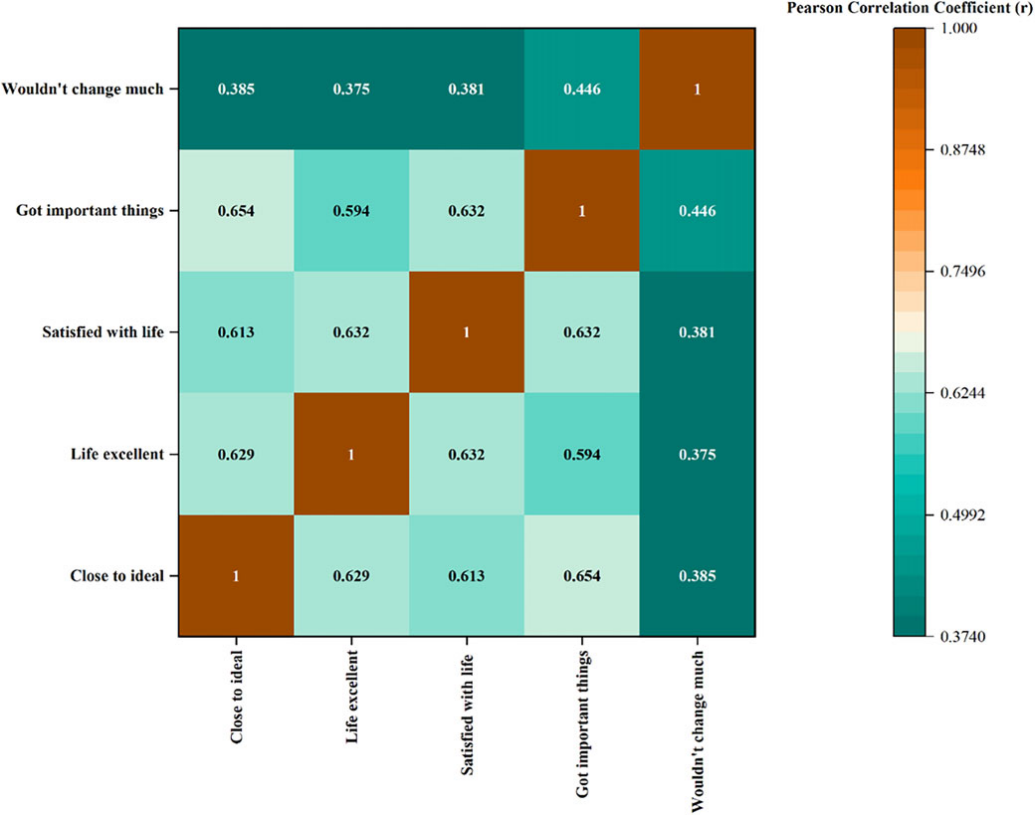
Heatmap visualization of SWLS item correlations.

The correlation matrix (Figure 2 and Table S2) revealed strong and statistically significant positive relationships among all five life satisfaction items (*p* < 0.01). The highest correlation was observed between ‘In most ways, my life is close to my ideal’ and ‘So far, I have gotten the important things I want in life’ (*r* = .654), indicating a close link between present life satisfaction and achievement of life goals. Similarly, ‘I am satisfied with my life’ was highly correlated with both ‘The conditions of my life are excellent’ (*r* = .632) and ‘So far, I have gotten the important things I want in life’ (*r* = .632), reflecting internal consistency across life satisfaction construct. Notably, the item ‘If I could live my life over, I would change almost nothing’ exhibited the weakest correlations with other items, though still statistically significant (*r* range = .375 to .446). This suggests that retrospective self-evaluation may represent a slightly distinct dimension of subjective well-being within this population.

### Life satisfaction and socio-demographical variables

Table 2 shows the means and standard deviations of SWLS scores by sex across marital statuses, age categories, family types, socioeconomic status and diet types. Comparisons using ANOVA or independent t-tests revealed a significant difference between males and females for marital status (*p* = 0.001), family type (*p* = 0.011) and diet type (*p* < 0.001). No significant sex differences were observed for age categories (*p* = 0.290) and socioeconomic status (*p* = 0.820). Males reported higher life satisfaction than females when married and cohabiting, living in joint families and following a non-vegetarian diet.

**Table 2.** SWLS scores by sex across socio-demographic and lifestyle factors

	Male		Female		
Variable	Mean ± SD	*N*	Mean ± SD	*N*	*p*-value
*Marital status*					
Never married	25.63 ± 6.40	65	25.56 ± 6.16	41	
Married & cohabiting	27.30 ± 6.21	145	23.75 ± 7.40	143	0.001*
Married, not cohabiting	22.50 ± 9.19	2	16.88 ± 3.64	8	
Divorced/widowed	14.00 ± —	1	15.00 ± —	1	
*Age*					
18–25	26.02 ± 5.66	53	25.45 ± 6.12	64	
26–35	27.30 ± 6.83	93	23.40 ± 7.58	92	
36–45	25.83 ± 6.80	47	22.26 ± 7.36	31	0.290
46–55	27.94 ± 4.79	17	23.25 ± 10.21	4	
56–65	25.33 ± 2.52	3	14.50 ± 0.71	2	
*Type of Family*					
Joint	26.79 ± 6.37	174	24.55 ± 6.79	159	0.011*
Nuclear	26.21 ± 6.40	39	20.29 ± 8.12	34	
*SES*					
Lower	24.33 ± 9.81	3	30.00 ± —	1	
Lower Middle	26.76 ± 6.26	173	23.67 ± 7.29	156	0.820
Upper Lower	26.58 ± 6.80	36	23.53 ± 6.88	32	
Upper Middle	23.00 ± —	1	29.50 ± 5.00	4	
*Diet Type*					
Vegetarian	23.42 ± 6.20	33	23.04 ± 6.69	71	<0.001*
Non-Vegetarian	27.28 ± 6.23	180	24.25 ± 7.48	122	

Values are presented as *Mean ± Standard Deviation (SD). N* denotes the number of participants by sex. *p*-values reflect comparisons using one-way ANOVA or independent *t*-tests are appropriate.

*Note*: Some subgroups (e.g., divorced/widowed or older age groups) included very few participants (*n* = 1–3). These data are shown for descriptive completeness only and were excluded from inferential conclusions.

Subgroups with extremely small sample sizes (e.g., divorced/widowed, older females and upper-status categories) were retained in the table for completeness but were not used to draw statistical inferences. These values are presented descriptively and should be interpreted with caution due to limited reliability.

Table 3 shows the percentiles corresponding to the total SWLS scores across age and sex groups showed that males consistently reported higher life satisfaction than females in all age categories, with mean scores ranging from 25.33 to 27.94 for males and 14.50 to 25.45 for females. Percentile distributions also reflected this trend, with higher median and upper percentile scores in males. However, small sample sizes in older age groups, particularly among females, limited the reliability of percentile estimates in those categories. Overall, these findings suggest a sex difference in life satisfaction, with males generally expressing greater satisfaction across the adult lifespan.

**Table 3.** SWLS percentiles by sex and age categories

	18–25		26–35		36–45		46–55		56–65	
Percentile	Male	Female	Male	Female	Male	Female	Male	Female	Male	Female
5th	15.0	16.0	14.0	10.7	14.0	9.8	14.0	11.0	23.0	14.0
10th	16.4	17.0	15.8	14.0	14.8	11.4	20.4	11.0	23.0	14.0
25th	22.5	20.0	22.0	17.0	20.0	16.0	25.5	13.25	23.0	14.0
50th	29.0	25.5	30.0	22.5	29.0	24.0	30.0	23.5	25.0	14.5
75th	30.0	30.75	32.5	30.0	30.0	29.0	30.0	33.0	–	–
90th	31.0	33.0	35.0	34.0	34.0	30.8	33.0	–	–	–
95th	34.3	34.0	35.0	35.0	35.0	32.6	–	–	–	–
*N*	53	64	93	92	47	31	17	4	3	2
Mean	26.02	25.45	27.30	23.40	25.83	22.26	27.94	23.25	25.33	14.50
SD	5.66	6.12	6.83	7.58	6.80	7.36	4.79	10.21	2.52	0.71

**Figure 3.**
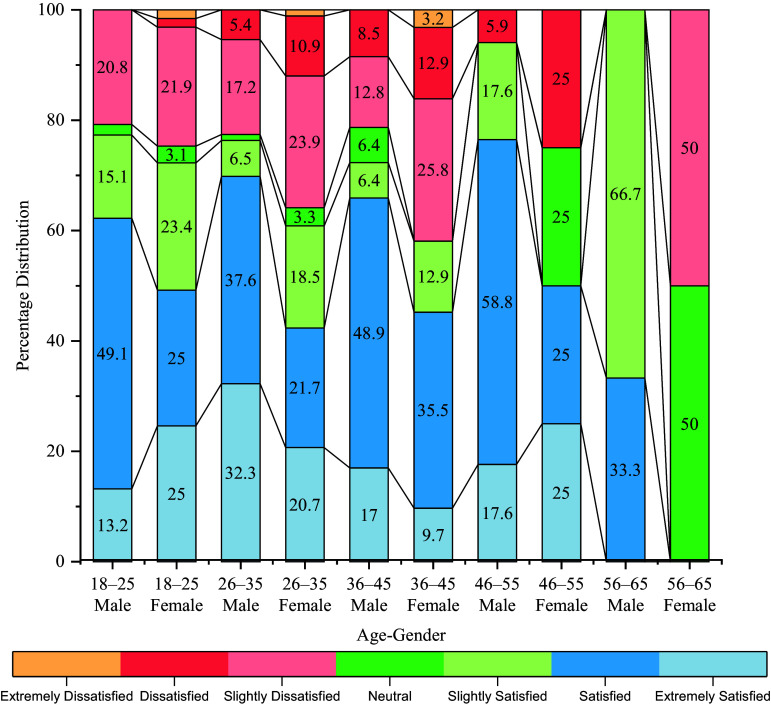
Distribution of satisfaction categories (SWLS) by age and gender.

Figure 3 and Table S3 shows notable age-and gender-related differences in life satisfaction. The ‘Satisfied’ category was most frequently reported, particularly among males aged 46–55 (58.8%), while females aged 56–65 reported no satisfaction at all. ‘Slightly satisfied’ responses were highest among males aged 56–65 (66.7%) and females aged 18–25 (23.4%). Conversely, older females (46–65 years) showed increased rates of dissatisfaction and neutrality, with up to 50% reporting low or neutral satisfaction. These trends highlight potential vulnerabilities in subjective well-being among older women, underscoring the need for gender-and age-responsive mental health interventions.

### Psychometric analysis of the SWLS

**Figure 4.**
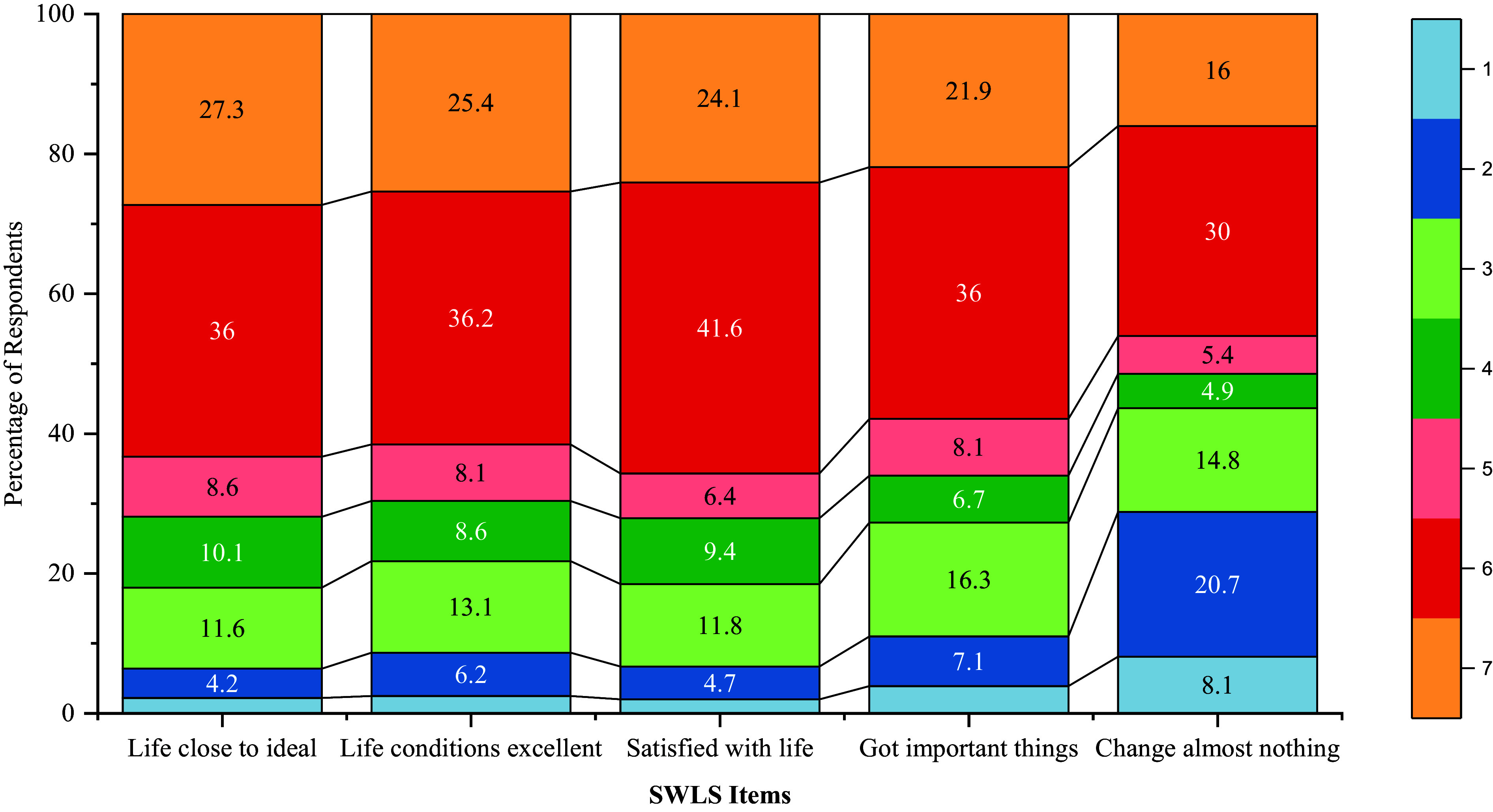
Response distribution across SWLS items.

In the present sample (*N* = 406), the SWLS showed strong psychometric properties and good construct validity. The mean SWLS score was 25.31 (SD = 6.58), indicating that, on average, respondents expressed a moderate to high level of life satisfaction with their lives. The internal consistency, Cronbach’s alpha, was .842, showing good reliability of the scale. The corrected item-total correlations ranged between .469 and .729, with items 1 to 4 contributing more strongly to consistency of the overall scale. Item 5, ‘If I could live my life over, I would change almost nothing’, had the lowest corrected item-total correlation (0.469). As can be seen from Figure 4, the values of skewness and kurtosis indicate that most items, except for item 5, have a slightly negative skew and a near-normal distribution; this item had a higher kurtosis, which suggests a flatter distribution. These findings add to the overall evidence supporting the reliability and validity of the scale for assessing subjective well-being. Complete psychometric statistics are available in Table S4.

A conceptual framework was developed in order to synthesize the observed patterns, depicting the interconnections among key variables influencing life satisfaction.

**Figure 5.**
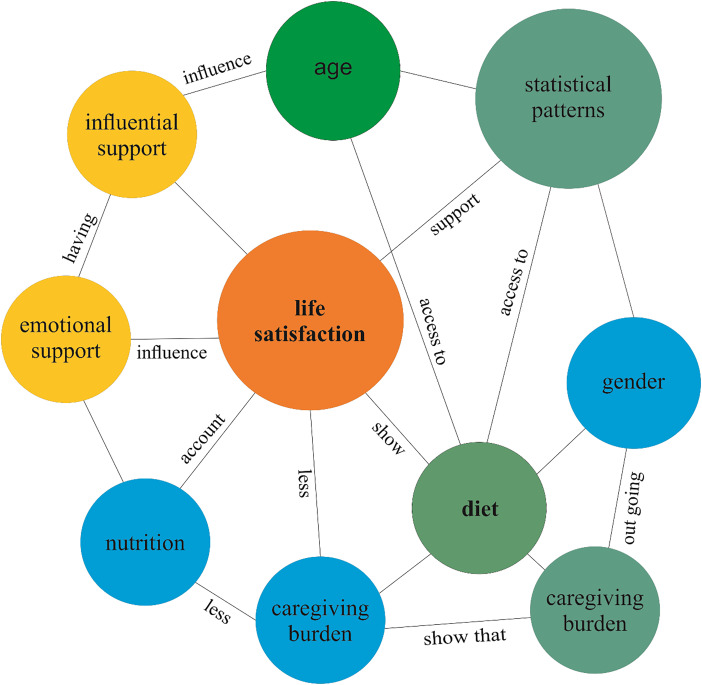
Conceptual framework showing interlinked socio-demographic, psychosocial, and lifestyle factors influencing life satisfaction in an urban slum. The figure shows patterns derived from statistical tables and highlights the interconnected role of gender, age, family type, and diet.

It is important to note that the conceptual framework (Figure 5) is interpretive rather than model-derived. It integrates observed statistical associations observed with theoretical insights to propose potential pathways that may shape life satisfaction. Although this framework describes demographic and lifestyle correlates, several contextual factors that were not measured are known to importantly influence subjective well-being in slum populations. Social discrimination, exposure to violence, migration status and security of housing tenure are recommended for inclusion in a future empirical model. The framework forms the basis for generating hypothesis and can be empirically validated in the future.

## Discussion

This study presents the first validation of the SWLS in an urban slum setting in India and reports complex patterns of subjective well-being in the context of extreme deprivation. Our findings show universal views of well-being by illustrating how poverty, social structures and cultural ways of coping influence how people evaluate their lives (Diener *et al.*, 1985). We relate these findings to wider debates within psychology, development studies and public policy.

In the slums of Gorakhpur, the SWLS revealed a strong internal reliability of *α* = 0.842, consistent with reports from other countries (Diener *et al.*, 1985; Pavot and Diener, 2008). However, one item, ‘If I could live my life over, I would change almost nothing’ performed less reliably. In the context of low-income settings, where people enjoy limited control over life choices, items based on personal autonomy or imagined alternatives may not fully capture lived experiences (Biswas-Diener and Diener, 2001). The implication, therefore, is well-being scales should be adapted to account for structural constraint and limited agency, especially in informal settlements where daily survival often overshadows long-term reflection (Ray, 2017; Espejo *et al.*, 2022).

It found lower life satisfaction among women, especially married and older women. This reflects the continuing impact of gender roles in slum communities, where women bear the invisible burden of unpaid work, caregiving and social isolation (Siddiqui, 2017; Desai *et al.*, 2004). Married women face additional burdens from household responsibilities and are further hindered by limited mobility. Older women, most of whom are widowed or economically dependent, reported the least satisfaction, reflecting lifelong impacts of gender-based inequality (Desai *et al.*, 2004; Thomas *et al.*, 2021). These patterns illustrate a need for measures of well-being that reach beyond household-level averages into differences within families. Scores for life satisfaction were higher for people in joint families than in nuclear ones. Responsibility is shared, emotional support exists and resources are pooled to shield them from economic stress (Howell and Howell, 2008). Such policies disturb these family structures; for instance, resettlement plans promoting nuclear living undermine significant sources of support for the urban poor (Desai *et al.*, 2004). This calls for a more sensitive approach to housing and urban planning, recognizing the protective role of extended families.

Respondents with non-vegetarian consumptions reported higher life satisfaction. This could be for many reasons. First, non-vegetarian diets are normally rich in proteins and other essential nutrients, which have been linked to better mental health outcomes (Huang and Zhao, 2020). Second, non-vegetarian meals might connote relief, variety, or celebration in food-insecure households, hence helping to raise positive emotions. Third, in certain cultural contexts, non-vegetarian food may be imbued with social value or associated with higher socioeconomic status, which could affect perceptions of satisfaction (Appadurai, 1981). Fourth, dietary choices may reveal something about wider household dynamics and decision-making power that is itself affecting well-being (Thakur *et al.*, 2018). Aside from the nutritional reasons, this association likely reflects structural and cultural determinants of well-being. In India, non-vegetarian diets are characteristics of relatively higher purchasing capacity, less strict dietary norms and greater social mobility. By implications, the positive association between life satisfaction and non-vegetarian consumption may be a proxy for relative affluence, urban exposure and social inclusiveness rather than a direct dietary effect (Biswas-Diener and Diener, 2001; Kumar *et al.*, 2021). Given these intersecting influences, caution is warranted when interpreting the association between diet type and life satisfaction. It is likely confounded by factors such as socioeconomic status, religion and caste, which may independently shape both dietary choices and subjective well-being. For this reason, this finding should be regarded as an exploratory association or hypothesis for future research rather than evidence of a causal relationship. Such an interpretation aligns with socioecological perspectives, which suggest that subjective well-being among low-income populations is shaped by complex interactions between economic means, social identity and access to diverse life experiences. Further research would be required to tease apart the relative contribution of nutritional, economic and socio-cultural factors in this association.

Notwithstanding economic deprivation, many respondents reported moderate life satisfaction. This resonates with the general trend in deprived communities, where individuals utilize various coping mechanisms, such as spiritual acceptance, comparison with less fortunate groups, or shared struggles, to maintain a semblance of dignity (Biswas-Diener and Diener, 2001; Veenhoven, 2005). Resilience, however, must not be confused with genuine well-being. Rather, this attests to the need for policies that address both material needs and emotional needs, taking full account of peoples capacity for adaptation without suppressing their unfulfilled aspirations. Overlapping demographic, social and lifestyle conditions shape life satisfaction, as is elaborated through the conceptual framework in Figure 5. The combination of joint families and non-vegetarian diet among men is associated with higher well-being.

This conceptual model is best considered an interpretive synthesis, reflecting the multidimensional nature of well-being among slum residents. Future studies with structural equation modelling or longitudinal data might empirically test and further refine these pathways.

## Limitations

This study had a cross-sectional design and thus any changes over time cannot be followed. Longitudinal research will be required in order to investigate how life satisfaction responds to an intervention, such as housing upgrading or some form of social programme. The SWLS might not fully capture the culturally specific ideas of well-being. Local terms such as *sukhi* (contentment) or *santosh* (satisfaction) may be more useful. These are better probed through qualitative methods. We did not explore household decision-making and domestic violence as factors that may account for gender differences in satisfaction. These aspects should be included in future research.

Moreover, the present study did not capture structural or psychosocial determinants of such outcomes, which may be related to exposure to violence, perceived stigma, or migration-related stressors known to shape well-being outcomes in disadvantaged communities.

In addition, a number of demographic subgroups represented in this study, like divorced/widowed and older participants, were very small. Therefore, findings related to these groups should be considered descriptive, rather than inferential, in nature and interpreted guardedly.

The fact that participants had to have an Android phone and be available for follow-up communication due to the inclusion criteria may indicate some degree of sampling bias in the study. While such a criterion conveniently facilitates operational logistics, the approach may have excluded households without mobile access, thus potentially limiting the representation of the most marginalized residents.

Moreover, the present analysis was not able to include several important independent variables like education, employment status and food security. These factors are known to affect subjective well-being and may combine with gender, family structure and dietary patterns in complex ways. We recommend that such dimensions be included in future studies to offer a more complete analysis of life satisfaction in low-income urban settings. We were also unable to include food security, so it is uncertain whether the diet-related findings reflect nutrition, income, or cultural practices.

Moreover, since dietary preferences in India are strongly shaped by socioeconomic position, religion and caste identity, the observed association between diet type and life satisfaction should be interpreted as exploratory rather than causal. In fact, this relationship is likely confounded by some unmeasured structural and cultural variables, which need further multivariate testing in future studies.

The present research was conducted on residents in one urban area and consisted of a predominantly Hindu population, which limits the generalizability of findings to other religious, cultural, or regional contexts across India. More comparative studies across different urban settings are needed to confirm whether the observed patterns hold in more diverse populations.

Because participation required basic phone access for follow-up communication, the sample may underrepresent households without mobile connectivity. This has been recognized as a potential source of selection bias that needs consideration in generalizing findings to the broader slum population. Community-based sampling in further research, especially without technological prerequisites for participation, will enhance the representativeness of the results.

## Conclusion

This study validates the use of the SWLS in slum communities while also showing its limits in capturing the full picture of well-being. We argue that policies for the urban poor must consider not only income and housing, but also social relationships, gender roles and psychological resilience. True development means respecting both practical needs and lived dignity of marginalized people. Well-being should be measured not only by what people have, but also by how they feel, cope and hope in the face of adversity.

## Policy implications

1. Integration of psychosocial screening into National Urban Health Mission (NUHM) urban primary health systems: Facilities for mental well-being assessments, including SWLS should be integrated into regular services at Urban Primary Health Centres (UPHCs) under NUHM. This recommendation directly stems from key finding of this paper regarding gender and age-based differences in life satisfaction among slum residents.
2. Develop gender-sensitive Mental Health Interventions (NMHP): NMHP should particularly focus on community-based psychosocial support for married, caregiving and elderly women group, which has been identified to show lower life satisfaction. Cultural adaptation of different forms of counselling and peer-support initiatives can reduce stress and social isolation among these groups.
3. Preserve Multigenerational Living in Urban Housing Schemes (PMAY-U, NHP): The study found higher life satisfaction among joint-family households, underscoring social and emotional benefits of shared living arrangements. Urban housing programmes such as PMAY-Urban should promote co-housing and multigenerational designs to retain these protective social structures during slum redevelopment.
4. Link Nutrition and Mental Health through Integrated NHM Initiatives: Rather than referring specifically to POSHAN Abhiyaan, recommendation now emphasizes broader NHM-based nutrition–mental health linkages. Given observed association between diet and well-being, nutrition-sensitive mental health promotion (e.g., food security, balanced diet education) can improve both physical and emotional outcomes in food-insecure adults.
5. Contextualize well-being indicators for National Monitoring (NHP, NMHS): Current national indicators may overlook cultural nuances of well-being. The National Health Policy (NHP) and National Mental Health Survey (NMHS) should adopt localized, context-sensitive measure of life satisfaction reflecting social, cultural and economic realities of marginalized urban communities.

## Supporting information

Venkatesh et al. supplementary material Venkatesh et al. supplementary material

## Data Availability

Available upon request from the corresponding author.

## Supplementary material

The supplementary material for this article can be found at https://doi.org/10.1017/S1463423626100991
