# Supplementary material for: Life satisfaction and subjective well-being in urban slums of Gorakhpur, India: psychometric validation of the satisfaction with life scale (SWLS) and socio-demographic assessment
Source: Prim Health Care Res Dev. 2026 Mar 6;27:e35. doi: 10.1017/S1463423626100991 (PMC12979020; doi:10.1017/S1463423626100991)
Supplement: Venkatesh et al. supplementary material [file S1463423626100991sup001.docx]

**Supplementary**

**Table S1.** Profile of slums

| Indicator | Gorakhpur City / Metropolitan Region |
| --- | --- |
| Estimated total population (2025) | 983,000 (city); 1,014,000 (metropolitan) |
| Total population (Census 2011) | 673,446 (city); 694,889 (metropolitan) |
| Urban population as % of district total (2011) | 18.84 % |
| Literacy rate (2011) | 83.91 % (total); male 88.67 %; female 78.65 % |
| Sex ratio (2011) | 903 females per 1,000 males |
| Total number of slums (city) | 8,056 |
| Population residing in slums (city) | 49,268 (7.3 % of urban population) |
| Major religions (2011) | Hindu 77.9 %; Muslim 20.6 %; Christian 0.7 %; Others < 1 % |
| No. of notified slums (Source – DUDA) | 110 |
| No. of non-notified slums (Source – UHI field database) | 56 |
| No. of slum households (Source – UHI field database) | 58,237 |
| Slums connected to sewerage network | 30 |
| Slums with primary school facility | 166 |
| Slums with primary healthcare facility | 166 |

Source: District Census Handbook (2011); Population Census India (2025 estimates); UHI field database (2023–24)

**Table S2.** Pearson’s *r* Correlation Matrix of Life Satisfaction Items

| Items | 1 | 2 | 3 | 4 | 5 |
| --- | --- | --- | --- | --- | --- |
| 1. In most ways, my life is close to my ideal | 1 | .629** | .613** | .654** | .385** |
| 2. The conditions of my life are excellent | .629** | 1 | .632** | .594** | .375** |
| 3. I am satisfied with my life | .613** | .632** | 1 | .632** | .381** |
| 4. So far, I have gotten the important things I want in life | .654** | .594** | .632** | 1 | .446** |
| 5. If I could live my life over, I would change almost nothing | .385** | .375** | .381** | .446** | 1 |

Note: Upper diagonal values are Pearson’s correlation coefficients.

Note: All correlations are significant at p < 0.01 (2-tailed).

**Table S3.** Percentage Distribution (%) of the total sample (N=406) in the different SWLS intervals according to gender and age

| SWLS Category | 18–25 years | | 26–35 years | | 36–45 years | | 46–55 years | | 56–65 years | |
| --- | --- | --- | --- | --- | --- | --- | --- | --- | --- | --- |
|  | **Male** | **Female** | **Male** | **Female** | **Male** | **Female** | **Male** | **Female** | **Male** | **Female** |
| Extremely satisfied | 13.2% | 25.0% | 32.3% | 20.7% | 17.0% | 9.7% | 17.6% | 25.0% | 0.0% | 0.0% |
| Satisfied | 49.1% | 25.0% | 37.6% | 21.7% | 48.9% | 35.5% | 58.8% | 25.0% | 33.3% | 0.0% |
| Slightly satisfied | 15.1% | 23.4% | 6.5% | 18.5% | 6.4% | 12.9% | 17.6% | 0.0% | 66.7% | 0.0% |
| Neutral | 1.9% | 3.1% | 1.1% | 3.3% | 6.4% | 0.0% | 0.0% | 25.0% | 0.0% | 50.0% |
| Slightly dissatisfied | 20.8% | 21.9% | 17.2% | 23.9% | 12.8% | 25.8% | 0.0% | 0.0% | 0.0% | 50.0% |
| Dissatisfied | 0.0% | 1.6% | 5.4% | 10.9% | 8.5% | 12.9% | 5.9% | 25.0% | 0.0% | 0.0% |
| Extremely dissatisfied | 0.0% | 1.6% | 0.0% | 1.1% | 0.0% | 3.2% | 0.0% | 0.0% | 0.0% | 0.0% |

**Table S4.** Percentage distribution (%) for each item of the Satisfaction With Life Scale (SWLS) and its psychometric properties (N = 406)

| Item | 1 | 2 | 3 | 4 | 5 | 6 | 7 | Mean | SD | α | Corrected Item-Test Corr. | Kurtosis | Skewness |
| --- | --- | --- | --- | --- | --- | --- | --- | --- | --- | --- | --- | --- | --- |
| In most ways my life is close to my ideal | 2.2% | 4.2% | 11.6% | 10.1% | 8.6% | 36.0% | 27.3% | 5.36 | 1.62 | - | .709 | -0.172 | -0.924 |
| The conditions of my life are excellent | 2.5% | 6.2% | 13.1% | 8.6% | 8.1% | 36.2% | 25.4% | 5.24 | 1.70 | - | .688 | -0.480 | -0.834 |
| I am satisfied with my life | 2.0% | 4.7% | 11.8% | 9.4% | 6.4% | 41.6% | 24.1% | 5.35 | 1.60 | - | .700 | -0.135 | -0.966 |
| So far, I have gotten the important things I want in life | 3.9% | 7.1% | 16.3% | 6.7% | 8.1% | 36.0% | 21.9% | 5.03 | 1.80 | - | .729 | -0.790 | -0.700 |
| If I could live my life over, I would change almost nothing | 8.1% | 20.7% | 14.8% | 4.9% | 5.4% | 30.0% | 16.0% | 4.33 | 2.08 | - | .469 | -1.538 | -0.170 |
| Total SWLS | – | – | – | – | – | – | – | 25.31 | 6.58 | .842 | – | – | – |
